# LncRNA SPRY4‐IT1 regulates breast cancer cell stemness through competitively binding miR‐6882‐3p with TCF7L2

**DOI:** 10.1111/jcmm.14786

**Published:** 2019-11-17

**Authors:** Xinyue Song, Xiaoxue Zhang, Xinnan Wang, Lianze Chen, Longyang Jiang, Ang Zheng, Ming Zhang, Lin Zhao, Minjie Wei

**Affiliations:** ^1^ Department of Pharmacology School of Pharmacy China Medical University Shenyang China; ^2^ Liaoning Key Laboratory of Molecular Targeted Anti‐Tumor Drug Development and Evaluation China Medical University Shenyang China; ^3^ Department of Medical Imaging Cancer Hospital of China Medical University Shenyang China; ^4^ Department of Breast Surgery The First Affiliated Hospital of China Medical University Shenyang China

**Keywords:** breast cancer, miR‐6882‐3p, SPRY4‐IT1, stem cells, TCF7L2

## Abstract

SPRY4‐intronic transcript 1 has been found in several kinds of cancers, but the role of SPRY4‐IT1 in breast cancer stem cells has not been studied. We investigated whether SPRY4‐IT1 is involved in the promotion of breast cancer stem cells (BCSCs). We used qRT‐PCR to detect the expression of SPRY4‐IT1 in MCF‐7 cells and MCF‐7 cancer stem cells (MCF‐7 CSCs). The effects of SPRY4‐IT1 on the proliferation and renewal ability of breast cancer cells were investigated by in vitro and in vivo assays (ie in situ hybridization, colony formation assay, sphere formation assay, flow cytometry assay, western blotting, xenograft model and immunohistochemistry). The mechanism of SPPRY4‐IT1 as a ceRNA was studied by a dual‐luciferase reporter assay and bioinformatic analysis. In our study, SPRY4‐IT1 was up‐regulated in MCF‐7 CSCs compared with MCF‐7 cells, and high SPRY4‐IT1 expression was related to reduced breast cancer patient survival. Furthermore, SPRY4‐IT1 overexpression promoted breast cancer cell proliferation and stemness in vitro and in vivo. In addition, SPRY4‐IT1 knockdown suppressed BCSC renewal ability and stemness maintenance in vivo and in vitro. The dual‐luciferase reporter assays indicated that SPRY4‐IT1 as a sponge for miR‐6882‐3p repressed transcription factor 7‐like 2 (TCF7L2) expression. Taken together, these findings demonstrated that SPRY4‐IT1 promotes proliferation and stemness of breast cancer cells as well as renewal ability and stemness maintenance of BCSCs by increasing the expression of TCF7L2 through targeting miR‐6882‐3p.

## 1 INTRODUCTION

Breast cancer was the most common cancer in females in 2018 with an incidence of 24.2% of all cancers in females. In addition, breast cancer caused 15% all cancer deaths of females in 2018.^1^ Increasing evidence has shown that heterogeneous tumour cell clusters make up the bulk of cancer tumours and that the tumour cell clusters originate from cells that have stem cell characteristics, namely cancer stem cells (CSCs).^2^ Cancer stem cells have abilities of multilineage differentiation, self‐renewal, high carcinogenic effect and chemoradiotherapy. In addition, CSCs mainly cause metastatic lesions and growth of tumours.^3^

Breast cancer stem cells (BCSCs) have the characteristics mentioned above. In 2003, Al‐Hajj and colleagues first found that breast cancer can be derived from BCSCs, and they also identified the surface markers of BCSCs to be CD44+/CD24−.^4^ Accumulating evidence has indicated that BCSCs are the major cause for metastasis, drug resistance and tumour recurrence. Thus, it is important to explore new treatments targeting BCSCs.

Long noncoding RNAs (lncRNAs) are transcript RNAs longer than 200 nucleotides without protein‐coding potential.^5^ LncRNAs are involved in physiological and pathological progresses, including embryonic development, organ formation, X chromatin inactivation and tumourigenesis.^6^, ^7^ LncRNAs play a key role in many diseases, especially in tumours,^8^ and they recruit transcription factors and regulate gene expression. LncRNAs also interact with messenger RNAs and influence the stability of mRNAs.^9^ SPRY4‐intronic transcript 1 (SPRY4‐IT1) is a type of lncRNA derived from an intron region within the SPRY4‐IT1 gene located at 5q31.3.^10^ Khaitan et al first found that SPRY4‐IT1 plays a key role in apoptosis and invasion in melanoma.^11^ SPRY4‐IT1 promotes cell growth, tumour proliferation and inhibits cell apoptosis in pancreatic ductal adenocarcinoma,^10^ cholangiocarcinoma,^12^ ovarian cancer,^13^ bladder cancer,^14^ hepatocellular carcinoma,^15^ oesophageal squamous cell carcinoma^16^ and breast cancer.^17^ However, Sun et al reported that overexpression of SPRY4‐IT1 inhibits non–small‐cell lung cancer cell proliferation and metastasis.^18^ Although SPRY4‐IT1 promotes the development of breast cancer,^12^ it is unclear if SPRY4‐IT1 influences the stemness of breast cancer cells.

In this study, we focused on identifying the effect of SPRY4‐IT1 and the underlying cellular and molecular mechanisms of SPRY4‐IT1 in BCSCs. We found that SPRY4‐IT1 was up‐regulated in CD44+/CD24− MCF‐7 cells compared to MCF‐7 parental cells and that SPRY4‐IT1 promoted self‐renewal and proliferation of MCF‐7 cells. Dual‐luciferase reporter analysis revealed that miR‐6882 was directly bound to SPRY4‐IT1 and TCF7L2, thereby down‐regulating their expression. These results indicated that SPRY4‐IT1 promotes the stemness of breast cancer cells by targeting miR‐6882 to regulate the activity of the Wnt/β‐catenin signalling pathway.

## 2 MATERIALS AND METHODS

### 2.1 Cell culture

The MCF‐7 and T47D breast cancer cell lines were obtained from ATCC (VA, USA) in 2017. All human cell lines have been authenticated using STR profiling. There were no mycoplasma contaminations in the cell lines. All human cell lines have been authenticated using STR profiling. There were no mycoplasma contaminations in the cell lines. MCF‐7 and T47D cell lines were cultured in high‐glucose (4.5 mg/mL) DMEM (HyClone) supplemented with 10% foetal bovine serum (FBS, HyClone), 100 mg/mL penicillin (Invitrogen) and 100 U/mL streptomycin (Invitrogen). MCF‐7 and T47D cell lines were maintained at 37°C in a 5% CO_2_ and 95% air incubator.

According to the induction technique of breast cancer MCF‐7 CSCs and T47D CSCs in our research group,^19^ MCF‐7 CSCs and T47D CSCs were incubated in DMEM‐F12 (HyClone) containing 2% B27 (Invitrogen), 20 μg/L EGF (Peprotech), 10 μg/L bFGF (Peprotech) at 37°C in a 5% CO_2_ and 95% air incubator.

### 2.2 In situ hybridization

RNA enzymes in sections were removed by 1 mL/L DEPC‐treated water and APES glue. Xylene was used to dewax the sections, and the sections were then rehydrated in a graded alcohol series. Endogenous peroxidase activity was blocked by 3% hydrogen peroxide, and mRNA was exposed by 3% fresh citric acid diluted pepsin. Sections were then incubated with 20 μL of digoxin‐labelled oligonucleotide probe and hybrid liquid at 37°C overnight. Blocking solution, biotinylated rat anti digoxin and SABC were then sequentially added according to the manufacturer's protocol of the lncRNA ISH Kit (Boster). DAB reagent was used to visualize the sections.

### 2.3 Tumour sphere formation assay

MCF‐7 and T47D CSCs (2 × 10^3^/well) were grown in serum‐free DMEM‐F12 supplemented with 10 μg/L bFGF, 20 μg/L EGF and 2% B27 in ultra‐low adhesion plates (Corning). Two weeks later, spheres larger than 100 μm were counted by an inverted microscope (Nikon TE2000‐U), and images were acquired.

### 2.4 Colony formation assay

MCF‐7 and T47D cells (2 × 10^3^) were seeded into 6‐cm Petri dishes. After 14 days of culture at 37 ℃ in 5% CO_2_, cells were washed with PBS, fixed in paraformaldehyde for 15 minutes and stained with 0.5% crystal violet for 15 minutes. Images were acquired, and the colonies were counted.

### 2.5 Cell transfection and virus infection

For transient transfection, miR‐6882 mimic and miR‐6882 inhibitor (RIOBOBIO) were transfected into MCF‐7 and T47D cells using Lipofectamine 3000 (Invitrogen) according to the manufacturer's protocol. For lentiviral transfection of shRNA knockdown analysis, lentiviral vectors (GV248) were purchased from Genechem Co., Ltd. SPRY4‐IT1‐RNAi (sequences shown in Table S1) was cloned into the hU6‐MCS‐Ubiquitin‐EGFP‐IRES‐puromycin vector (Genechem). MCF‐7 CSCs and T47D CSCs were transduced with shRNAs using SPRY4‐IT1 (sh‐SPRY4‐IT1) lentiviral transduction particles (MOI = 20) in the presence of 5 µg/mL polybrene (Genechem). For cDNA knock‐in analysis, lentiviral vectors (GV502) were purchased from Genechem. SPRY4‐IT1 cDNA was cloned into the (polyA‐MCS‐UBI) RV‐SV40‐EGFP‐IRES‐puromycin vector (Genechem). MCF7 CSCs and T47D CSCs were transduced with SPRY4‐IT1 (SPRY4‐IT1‐cDNA) lentiviral transduction particles (MOI = 20) (Genechem). Twenty hours after transfection, the medium containing virus was replaced with fresh culture medium. Stably transfected cells were selected by adding puromycin (1 μg/mL) into the medium. This selection was repeated 2 to 3 times until green fluorescent protein (GFP) was observed in all cells under a fluorescence microscope (Nikon TE2000‐U).

### 2.6 Flow cytometry assay

MCF‐7 cells, T47D cells, MCF‐7 CSCs and T47D CSCs were digested with 0.25% trypsin, and they were then stained with anti‐CD44‐APC (Biolegend) (1.25 μL/test) and anti‐CD24‐PE (Biolegend) (5 μL/test) or negative controls at 4°C for 30 minutes. After staining, cells were washed three times with PBS and suspended in 300 μL of PBS. Flow cytometry analysis was performed on a MACSQuant^TM^ Flow Cytometer (Miltenyi Biotec).

### 2.7 Dual‐luciferase reporter assay

The full‐length SPRY4‐IT1 sequences of the wild‐type (WT) and mutant (MUT) miRNA‐binding sites were purchased from Genechem. SPRY4‐IT1 WT and SPRY4‐IT1 MUT were transfected into MCF‐7 cells along with miR‐6882 mimic or NC mimic. Similarly, the binding sites for miR‐6882‐3p in the 3’‐untranslated region (3’‐UTR) sequence of TCF7L2 were purchased from Genechem. TCF7L2 3’‐UTR WT and TCF7L2 3’‐UTR MUT were transfected into MCF‐7 cells along with miR‐6882 mimic or NC mimic. Luciferase activity was measured by the dual‐luciferase reporter assay system (Promega) according to the manufacturer's instructions. Assays were performed in triplicates.

### 2.8 Data extraction and analysis from TCGA

MiRNAs with complementary sequences to SPRY4‐IT1 were searched for using online miRDB (http://www.mirdb.org/) and SEGAL (https://genie.weizmann.ac.il/index.html) datasets. The RNAhybrid website (https://bibiserv.cebitec.uni-bielefeld.de/rnahy‐ brid/) was used to predict the binding energy of SPRY4‐IT1 and miR‐6882. DIANA Tools (http://www.microrna.gr) and miRDB (http://mirdb.org/miRDB/index.html) were used to predict the target gene of miR‐6882‐3p. RNA‐Seq data were downloaded from TCGA‐breast cancer (TCGA‐BRCA) (https://cancergenome.nih.gov/). The different expression levels of SPRY4‐IT1 in the two groups were divided according to the expression of CD24 and CD44, and the differences in HIF‐1α, HIF‐2α and OCT4 expression levels between SPRY4‐IT1 low and high expression were analysed. Kaplan‐Meier survival analysis for the correlation between SPRY4‐IT1 expression and survival time of breast cancer patients was performed using Kaplan‐Meier Plotter (http://kmplot.com/analysis).

### 2.9 RNA extraction and qRT‐PCR analysis

Trizol reagent (CWBIO) was used to extract total RNA according to the manufacturer's instructions, and RNA was converted to complementary DNA (cDNA) using the qPCR RT kit (TOYOBO) and oligo (dT) primers. The method of miRNA extraction was similar to total RNA extraction. MiRNA was reversely transcribed into cDNA using the miRNA qRT‐PCR Starter kit (Riobobio). PCR primers for U6 and miR‐6882 were purchased from Riobobio. Quantitative real‐time PCR was performed using the SYBR Green PCR Mix kit (Takara). The detailed primer sequences are shown in Table S1. Results were normalized according to β‐actin mRNA or U6 miRNA expression levels. The results were expressed using the ∆∆CT (cycle threshold) method for quantification.

### 2.10 Western Blotting and Immunohistochemistry (IHC)

The NE‐PERTM Nuclear and Cytoplasmic Extraction Reagents kit (Thermo Scientific) was used to isolate and collect cytosolic and nuclear fractions following the manufacturer's protocol. Cells were lysed in radioimmunoprecipitation assay (RIPA) lysate buffer, and cell lysates were incubated on ice for 30 minutes. Cell supernatants were collected, and protein concentrations were determined using bicinchoninic acid (BCA) protein quantitation (Beyotime). SDS‐PAGE electrophoresis was performed on proteins from cell lysate proteins and transferred to PVDF membranes. Membranes were incubated with primary antibody overnight at 4℃. The primary antibodies and secondary antibodies are shown in Table S2. Proteins in membranes were visualized using an enhanced chemiluminescence kit (BOSTER).

For IHC, mouse tumour sections were dewaxed and rehydrated. The antigen was retrieved under high pressure using citrate buffer (pH = 6.0). The Ultra‐sensitive S‐P kit (Maixin‐Bio) was used to block endogenous peroxidase activity and reduce non‐specific reactivity. Sections were then incubated with primary antibodies (shown in Table S2) at 4°C overnight. Mouse tumour sections were then incubated with secondary antibody and streptomycin avidin‐peroxidase using the Ultra‐sensitive S‐P kit, and the sections were visualized with DAB reagent (Maixin‐Bio).

### 2.11 Xenograft model

To study the SPRY4‐IT1 stemness ability of MCF‐7 and MCF‐7 CSCs, cells (1 × 10^6^) were suspended in 100 μL of PBS and injected into mammary fat pads of 3‐ to 4‐week‐old female BALB/c(nu/nu) mice (Hua Fukang Biological Technologies Inc, Beijing). Mice were randomized into the following four groups (n = 6 per group): NC‐cDNA with MCF‐7; SPRY4‐IT1‐cDNA with MCF‐7; sh‐NC with MCF‐7 CSCs; and sh‐SPRY4‐IT1 with MCF‐7 CSCs. The tumour diameter and weights of mice were measured every other day. Tumours were removed from xenograft models and weighed until tumours appeared to collapse. Tumour volume (mm^3^) was measured using a digital calliper and calculated according to the following equation: (width)^2^ × (length/2). All mice were bred at pathogen‐free conditions in the Animal Centre of China Medical University. All animal studies were performed according to the National Institute of Health Guide for the Care and Use of Laboratory Animals.

### 2.12 Statistical analysis

Quantitative data were expressed as the means ± SD of at least three independent experiments. Graphpad Prism 7.0 (GraphPad Software) was used to evaluate all experimental values. Student's independent *t* test was used to perform statistical analysis between two experimental groups, while analysis of variance (ANOVA) was used to perform analyses among three experimental groups. *P* value < .05 was considered statistically significant in all cases.

## 3 RESULTS

### 3.1 BCSCs express high SPRY4‐IT1 level compared to non‐CSC cells

Because the induction technique of breast cancer MCF‐7 CSCs has matured in our research group and has been published in related articles,^19^, ^20^ this paper briefly verified the induction of breast cancer MCF‐7 CSCs.

**Figure 1:**
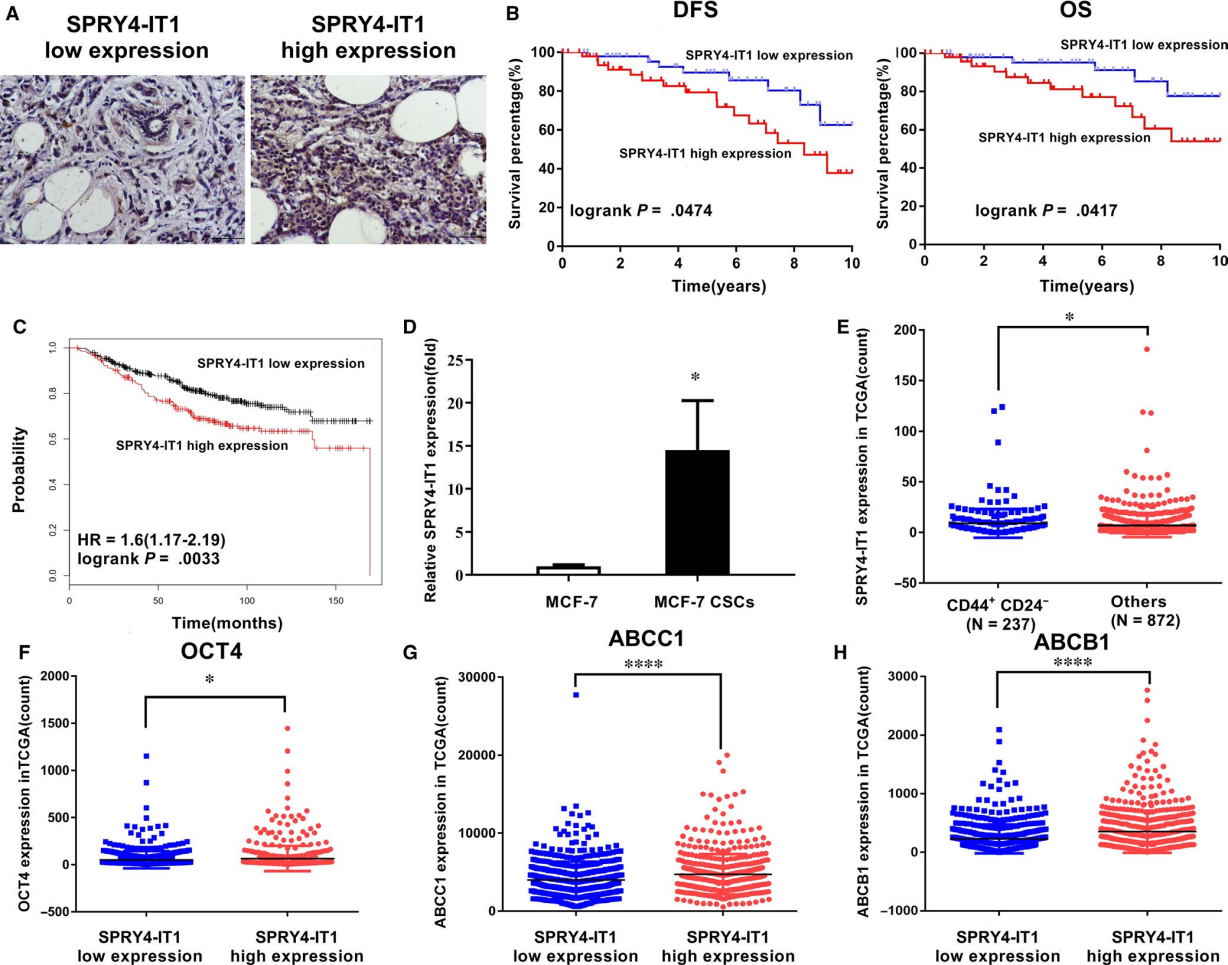
SPRY4‐IT1 is up‐regulated in breast cancer stem cells and is correlated with prognosis. A, SPRY4‐IT1 expression in breast cancer patients by in situ hybridization. Original magnification, ×200. Scale bars, 100 μm. B, Kaplan‐Meier survival analysis of breast cancer patients’ overall and disease‐free survival based on SPRY4‐IT1 expression in our cohort (n = 101, *P* = .0474 and *P* = .0417, respectively). C, Kaplan‐Meier survival analysis of breast cancer patients’ overall survival based on SPRY4‐IT1 expression by Kaplan‐Meier Plotter (*P* = .0033). D, mRNA expression level of SPRY4‐IT1 in MCF‐7 cells and MCF‐7 CSCs detected by qRT‐PCR. E, SPRY4‐IT1 mRNA expression level in the CD44+/CD24‐ group and other groups from RNA sequencing data from TCGA‐BRCA database. OCT4(F), ABCC1(G) and ABCG1(H) mRNA expression levels in the SPRY4‐IT1 low expression group and high expression group from RNA sequencing data from TCGA‐BRCA database. *P < .05, **P < .01, ***P < .001, ****P < .0001

In situ hybridization revealed that SRY4‐IT1 was expressed in the cytoplasm (Figure 1A). According to the expression of SPRY4‐IT1 in 101 samples from breast cancer patients from the First Affiliated Hospital of China Medical University (Figure 1A), we divided the breast cancer patients into two groups. The log‐rank test of the overall survival curves of these breast cancer patients showed that the high SPRY4‐IT1 expression group was significantly associated with worse OS and DFS compared to the low SPRY4‐IT1 expression group (Figure 1B). Moreover, the log‐rank test of the OS curves of breast cancer patients in Kaplan‐Meier plotter also identified that high SPRY4‐IT1 expression was significantly associated with worse OS compared to low SPRY4‐IT1 expression (Figure 1C). The expression of SPRY4‐IT1 in MCF‐7 cells and MCF‐7 CSC cells was detected by qRT‐PCR. The expression of SPRY4‐IT1 was significantly increased in MCF‐7 CSCs compared to MCF‐7 cells (Figure 1D). These results suggested that SPRY4‐IT1 is associated with MCF‐7 CSC characteristics.

Further, we downloaded RNA‐Seq data from breast cancer patients from TCGA‐breast cancer database and found that the expression level of SPRY4‐IT1 was significantly higher in patients with CD44+/CD24‐ breast cancer (n = 237) than those without CD44+/CD24− (n = 872) (Figure 1E). The OCT4 stemness marker was expressed at higher levels in breast cancer tissues with SPRY4‐IT1 high expression compared to tissues with low SPRY4‐IT1 expression (Figure 1F). In addition, drug resistance‐related genes, such as ATP‐binding cassette sub‐family C member 1 (ABCC1) and ATP‐binding cassette sub‐family B member 1 (ABCB1), were up‐regulated in the low SPRY4‐IT1 expression group compared to the high SPRY4‐IT1 expression group in TCGA‐BRCA database (Figure 1G,H). These results suggested that the expression of SPRY4‐IT1 may be associated with the stemness phenotype of BCSCs.

### 3.2 SPRY4‐IT1 influences cell stemness characteristics of MCF‐7 and T47D cells

**Figure 2:**
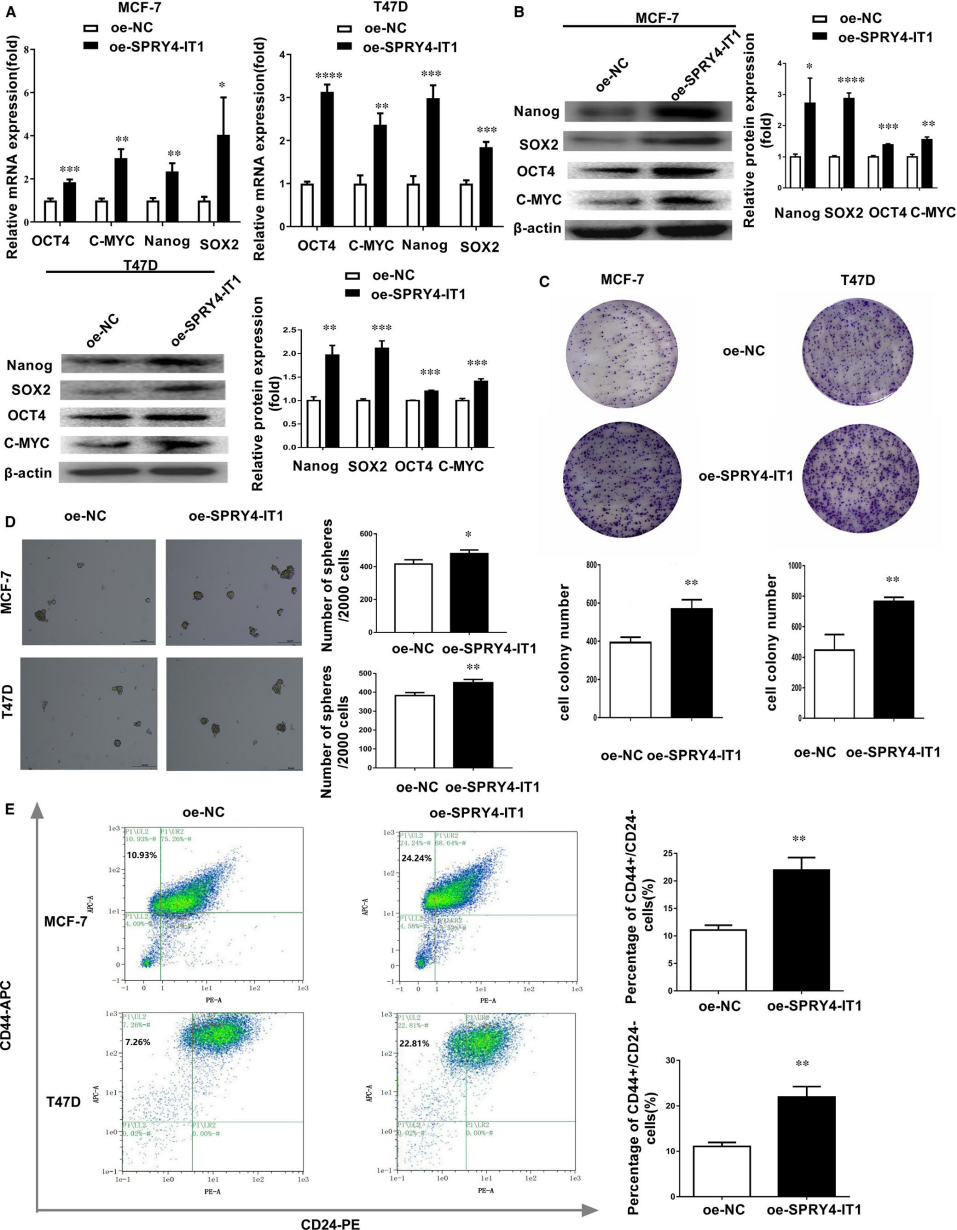
SPRY4‐IT1 promotes cell stemness characteristics of MCF‐7 and T47D cells. A, Expression of stemness markers (OCT4, C‐MYC, Nanog and SOX2) was detected by qRT‐PCR in MCF‐7 and T47D cells transfected with SPRY4‐IT1. B, Left: Expression of stemness markers (Nanog, SOX2, OCT4 and C‐MYC) was detected in SPRY4‐IT1‐overexpressing MCF‐7 and T47D cells by Western blotting. Right: Densitometric analysis of protein expression. C, MCF‐7 and T47D cells transfected with SPRY4‐IT1 were seeded in 6 cm Petri dishes. After 14 d, the number of colonies was counted. D, Sphere formation assays showed the mammosphere‐forming ability of MCF‐7 and T47D cells transfected with SPRY4‐IT1. Original magnification, ×40. Scale bars, 100 μm. E, Flow cytometry indicated the CD44+/CD24 − percentage in MCF‐7 and T47D cells transfected with SPRY4‐IT1. Data are presented as the mean ± SD of three independent experiments performed in triplicate. *P < .05, **P < .01, ***P < .001, ****P < .0001

We established MCF‐7 and T47D with SPRY4‐IT1 overexpression (oe‐SPRY4‐IT1) (Figure S1A). qRT‐PCR showed that the expression levels of cell stemness markers, including OCT4, C‐MYC, Nanog and SOX2, were increased in SPRY4‐IT1‐overexpressing MCF‐7 and T47D cells (Figure 2A). Western blot assay showed that Nanog, SOX2, OCT4 and C‐MYC expression levels were increased in SPRY4‐IT1‐overexpressing MCF‐7 and T47D cells (Figure 2B). Plate colony formation assay revealed that the number of colonies of oe‐SPRY4‐IT1 MCF‐7 and T47D cells was increased compared to negative controls (Figure 2C). Sphere formation assay indicated that MCF‐7 and T47D cells transfected with SPRY4‐IT1 had more spheres compared to negative controls (Figure 2D). Flow cytometry analysis revealed that the percentages of CD44+/CD24− cells were increased in SPRY4‐IT1‐overexpressing MCF‐7 and T47D cells (Figure 2E).

### 3.3 SPRY4‐IT1 influences cell stemness maintenance of MCF‐7 CSCs and T47D CSCs

**Figure 3:**
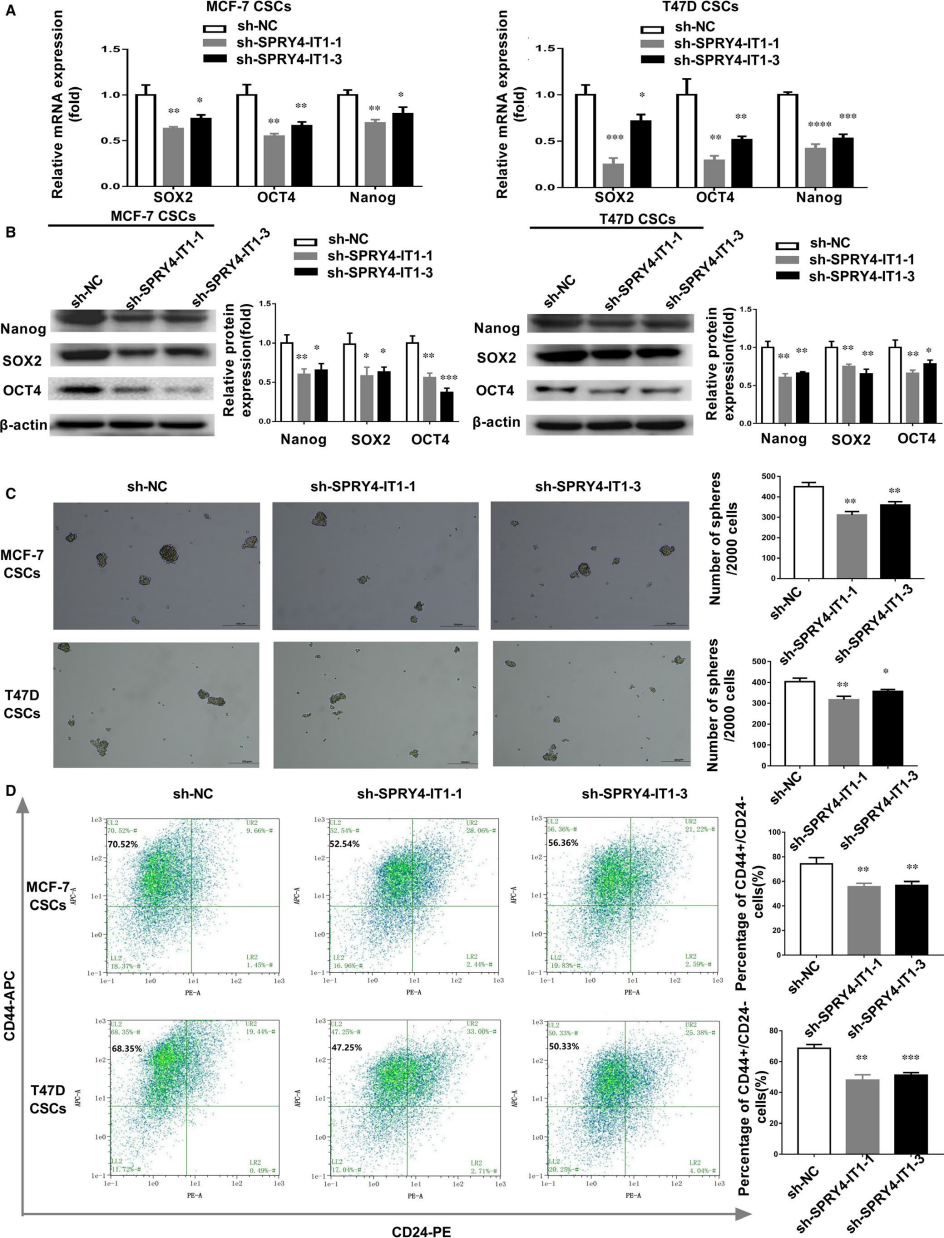
SPRY4‐IT1 affects cell stemness maintenance of MCF‐7 and T47D CSCs. A, Expression of stemness markers (SOX2, OCT4 and Nanog) was detected by qRT‐PCR in MCF‐7 and T47D CSCs transfected with two sh‐SPRY4‐IT1. B, Left: Expression of stemness markers (Nanog, SOX2 and OCT4) was detected in sh‐SPRY4‐IT1 MCF‐7 and T47D CSCs by western blotting. Right: Densitometric analysis of protein expression. C, Sphere formation assays showed the mammosphere‐forming ability of MCF‐7 and T47D CSCs transfected with sh‐SPRY4‐IT1. Original magnification, ×40. Scale bars, 100 μm. D, Flow cytometry indicated the CD44+/CD24 − percentage in MCF‐7 and T47D CSCs transfected with sh‐SPRY4‐IT1. Data are presented as the mean ± SD of three independent experiments performed in triplicate. *P < .05, **P < .01, ***P < .001, ****P < .0001

We established MCF‐7 and T47D CSCs in which SPRY4‐IT1 was stably knocked down (sh‐SPRY4‐IT1) (Figure S1B). SOX2, Nanog, and OCT4 mRNA expression levels were down‐regulated in SPRY4‐IT1‐deficient MCF‐7 and T47D CSCs (Figure 3A). Western blot assay showed that the protein expression levels of cell stemness markers, including Nanog, SOX2, and OCT4, were reduced in sh‐SPRY4‐IT1 MCF‐7 CSCs and T47D CSCs (Figure 3B). Next, a mammosphere formation assay was performed, and the sphere‐like structures with diameters greater than 100 μm were counted. The percentage of sphere‐forming cells was reduced after inhibiting the expression of SPRY4‐IT1 in MCF‐7 and T47D CSCs (Figure 3C). Flow cytometry analysis showed that percentages of CD44+/CD24− cells were down‐regulated in SPRY4‐IT1‐deficient MCF‐7 CSCs and T47D CSCs (Figure 3D). These results indicated that SPRY4‐IT1 influences breast cancer cell stemness maintenance.

### 3.4 SPRY4‐IT1 regulates the expression of TCF7L2 by miR‐6882‐3p

**Figure 4:**
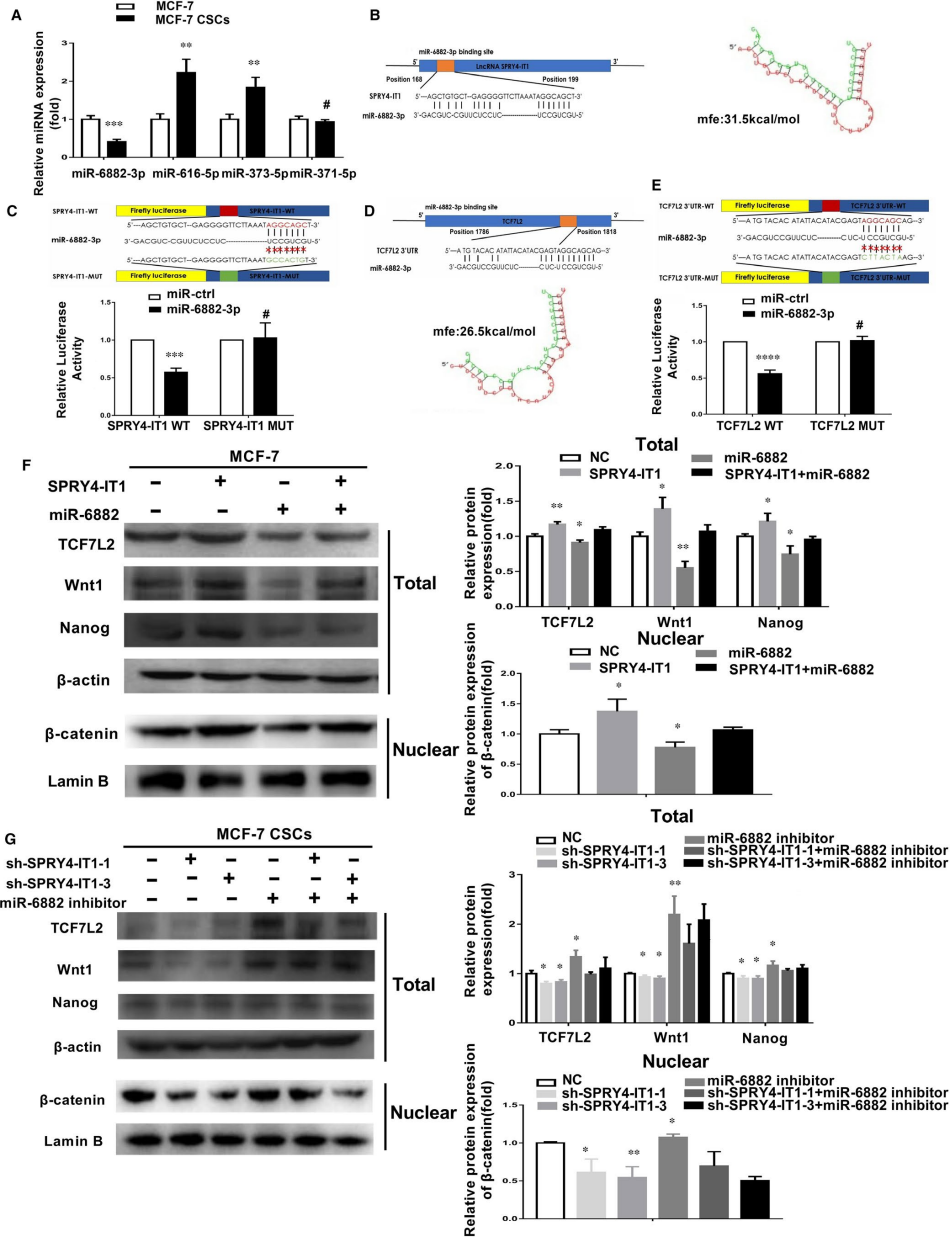
SPRY4‐IT1 modulates expression of TCF7L2 protein through sponging miR‐6882‐3p. A, qRT‐PCR was used to identify miRNAs that directly interacted with SPRY4‐IT1. B, The complementary binding of miR‐6882‐3p and SPRY4‐IT1 as well as predicted binding energy. C, Top: Complementary binding of miR‐6882‐3p and wild‐type/mutant SPRY4‐IT1. Bottom: Dual‐luciferase reporter assays indicated the interaction of miR‐6882‐3p and SPRY4‐IT1. D, Complementary binding of miR‐6882‐3p and TCF7L2 3’‐UTR as well as predicted binding energy. E, Top: Complementary binding of miR‐6882‐3p and wild‐type/mutant TCF7L2 3’‐UTR. Bottom: Dual‐luciferase reporter assays showed the combination of miR‐6882‐3p and TCF7L2 3’‐UTR. F Wnt1/β‐catenin signalling pathway‐related protein expression (TCF7L2, Wnt1, β‐catenin (Nuclear) and Nanog) was measured by western blotting after overexpression of SPRY4‐IT1 and transfection with miR‐6882‐3p or miR‐NC in MCF‐7 cells. G Wnt1/β‐catenin signalling pathway‐related protein expression (TCF7L2, Wnt1, β‐catenin (Nuclear) and Nanog) was measured by western blotting after knockdown of SPRY4‐IT1 and transfection with miR‐6882‐3p or miR‐NC in MCF‐7 CSCs. Data are presented as the mean ± SD of three independent experiments performed in triplicate. *P < .05, **P < .01, ***P < .001, ****P < .0001

LncRNA can be the molecular sponge of miRNA as a ceRNA.^21^ Using both datasets, we found that miR‐6882‐3p, miR‐616‐5p, miR‐373‐5p and miR‐371B‐5p interacted with SPRY4‐IT1. As shown in Figure 4A, miR‐6882‐3p expression was remarkably reduced in MCF‐7 CSCs compared to MCF‐7 cells. RNAhybrid showed that the predicted binding energy of miR‐6882‐3p and SPRY4‐IT1 is −31.5 kcal/mol, which suggested that they interacted (Figure 4B). The binding sites of miR‐6882‐3p in SPRY4‐IT1 are shown in Figure 4B. To identify if miR‐6882‐3p binds to the predicted target site of SPRY4‐IT1, we constructed SPRY4‐IT1 wild‐type and mutant luciferase reporter vectors according to the indicated binding site for miR‐6882‐3p (Figure 4C). Co‐transfection of SPRY4‐IT1 wild‐type vector (SPRY4‐IT1‐WT) with miR‐6882‐3p mimics rather than the mutant SPRY4‐IT1 vector (SPRY4‐IT1‐MUT) remarkably decreased luciferase activities in MCF‐7 cells (Figure 4C). qRT‐PCR demonstrated that SPRY4‐IT1 overexpression reduced miR‐6882‐3p expression in MCF‐7 cells (Figure S1C) and that knockdown of SPRY4‐IT1 remarkably increased miR‐6882 expression (Figure S1D). In addition, the expression of SPRY4‐IT1 also influenced stemness characteristics, including Nanog, CD44 and OCT4. SPRY4‐IT1 overexpression promoted MCF‐7 and T47D cell stemness characteristics (Figure S1E). SPRY4‐IT1 knockdown inhibited MCF‐7 and T47D CSC stemness maintenance (Figure S1F). Thus, these results indicated that SPRY4‐IT1 plays a role as a molecular sponge for miR‐6882‐3p and that the stemness promotion effect of SPRY4‐IT1 partially relies on sponging miR‐6882‐3p.

We then studied the targets of SPRY4‐IT1 ceRNA. To identify the target gene of miR‐6882‐3p, we used the mirDB database and DIANA database, and the results of these databases were combined. According to a previous study,^22^ the Wnt signalling pathway increases the number of BCSCs and is vital to the maintenance of BCSCs. In the present study, we found that miR‐6882‐3p binds to TCF7L2. Transcription factor 7‐like 2 (TCF7L2), also called TCF4, is an effector of the Wnt/β‐catenin signalling pathway^23^ and a key transcription regulator that forms a TCF7L2/β‐catenin complex with β‐catenin, which transcriptionally activates downstream factors in the Wnt pathway. The binding energy was predicted to be −26.5 kcal/mol by RNAhybrid, suggesting that they interact (Figure 4D). We subcloned the TCF7L2 mRNA 3’‐UTR region, including the predicted miR‐6882 recognition site, for wild‐type (TCF7L2 WT）and mutant (TCF7L2 MUT) luciferase reporter plasmids. MiR‐6882 decreased luciferase activity in the wild‐type vector compared to the mutant vector (Figure 4E). Moreover, TCF7L2 and Wnt1 mRNA expression was increased by inhibiting miR‐6882‐3p expression in MCF‐7 cells but was decreased in miR‐6882‐3p‐overexpressing MCF‐7 CSCs (Figure S1G‐H). These results suggested that TCF7L2 is a target gene of miR‐6882. Furthermore, we investigated if SPRY4‐IT1 affects the miR‐6882/TCF7L2 axis. Overexpression of SPRY4‐IT1 in MCF‐7 cells remarkably increased mRNA and protein expression of TCF7L2 and the Wnt1/β‐catenin pathway components (Figure 4F and Figure S1I). Translocation of β‐catenin from the cytoplasm to the nucleus is crucial for activation of the Wnt1/β‐catenin pathway. The protein expression of β‐catenin was increased in the nuclear fraction (Figure 4F), and the protein expression levels of TCF7L2 and Wnt1/β‐catenin (Wnt1 [Total] and β‐catenin [Nuclear]) pathway components were decreased by overexpressing miR‐6882 in MCF‐7 cells (Figure 4F). In contrast, knockdown of SPPRY4‐IT in MCF‐7 CSCs decreased the mRNA and protein expression levels of TCF7L2 and Wnt1/β‐catenin pathway (Wnt1 [Total] and β‐catenin [Nuclear]) components (Figure S1J and Figure 4G). In addition, inhibition of miR‐6882 in MCF‐7 CSCs increased the protein expression levels of TCF7L2 and Wnt1/β‐catenin pathway (Wnt1 [Total] and β‐catenin [Nuclear]) components (Figure 4G). These results revealed that SPRY4‐IT1 influences TCF7L2 expression by targeting miR‐6882, which promotes β‐catenin accumulation in the nucleus, thereby activating the Wnt1/β‐catenin pathway.

### 3.5 SPRY4‐IT1 enhances the stemness and self‐renewal capacity of BCSCs in vivo

**Figure 5:**
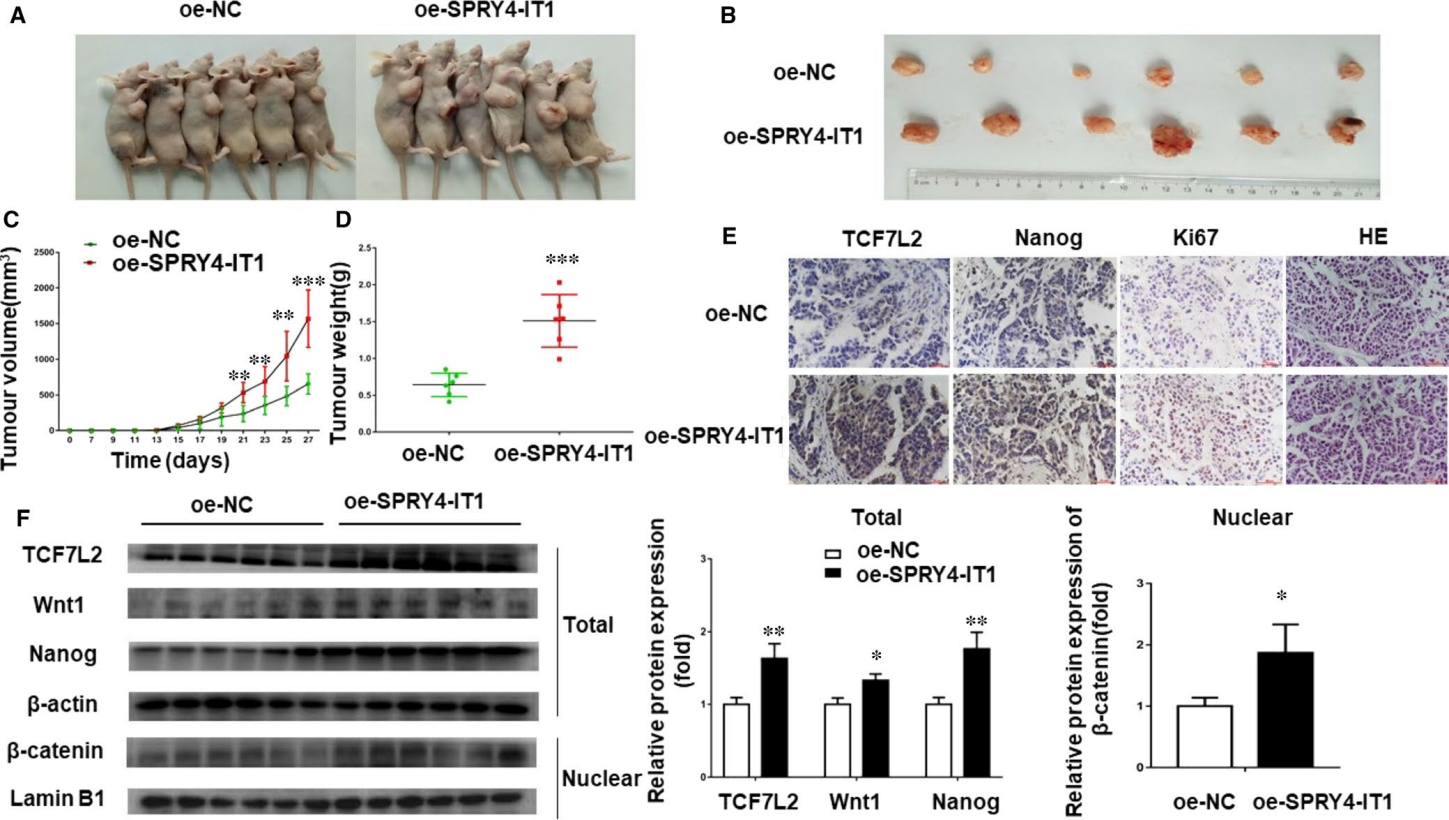
SPRY4‐IT1 enhances the stemness of BCSCs in vivo*.* A, Subcutaneous tumour from the SPRY4‐IT1 overexpression (oe‐SPRY4‐IT1) group and negative control group. B, Images of oe‐SPRY4‐IIT1 MCF7 tumour tissues. C, Average tumour volumes were measured in xenograft mice every two days. D, Images of average tumour weight at the end of indicated treatment. E, Immunohistochemistry analysis of TCF7L2, Nanog and Ki‐67 protein levels in tumour tissues formed from SPRY4‐IT1‐overexpressing cells or control cells. Original magnification, ×400. Scale bars, 50 μm. F, Wnt1/β‐catenin signalling pathway‐related protein expression (TCF7L2, Wnt1, β‐catenin (Nuclear) and Nanog) was measured by western blotting in oe‐NC and oe‐SPRY4‐IT1 groups. Data are presented as the mean ± SD of three independent experiments performed in triplicate. *P < .05, **P < .01, ***P < .001, ****P < .0001

To identify the effect of SPRY4‐IT1 on stemness promotion of MCF‐7 cells, MCF‐7 cells transfected with SPRY4‐IT1‐cDNA or NC‐cDNA were subcutaneously injected into nude mice (Figure 5A). Overexpression of SPRY4‐IT1 significantly increased the tumour size and weight (Figure 5B‐D). Western blot and IHC analyses demonstrated that the expression of the Nanog stemness marker was up‐regulated by SPRY4‐IT1 overexpression (Figure 5E,F), indicating promotion of stemness. The IHC staining results revealed that the expression of TCF7L2 was higher in the SPRY4‐IT1‐cDNA group compared to the NC‐cDNA group (Figure 5E). Furthermore, tumour sections were stained for Ki‐67 expression to assess the proliferation ability of xenograft tumours. The ability of proliferation was higher in the SPRY4‐IT1 overexpression group (Figure 5E). TCF7L2, Wnt1 and β‐catenin (nuclear) protein expression increased in tumours overexpressing SPRY4‐IT1 (Figure 5F). According to these results, we concluded that SPRY4‐IT1 promotes stemness of breast cancer cells and exerts its function through TCF7L2 in vivo.

Furthermore, the results of mice infected with sh‐SPRY4‐IT1 MCF‐7 CSCs and their negative controls were consistent with the above results (Figure S2).

## 4 DISCUSSION

Breast cancer is one of the most common malignant cancers worldwide and is the leading cause of cancer‐related death in women.^24^ Cancer stem cells are defined similarly to normal stem cells as cells that are capable self‐renewal, leading to multi‐linage differentiation of malignant tumour cells.^25^ Moreover, cancer stem cells may explain several phenomena of cancers, such as chemoradiation resistance and metastasis.^26^ With regard to clinical treatments, CSCs are less sensitive to antitumour drugs compared to tumour cells, contributing to the recurrence of cancers.^27^ Recent studies have shown that lncRNAs are important for CSC biological functions in many types of cancers.^28^ Yao et al found that lncRNA XIST is up‐regulated in glioblastoma CSCs and promotes stemness characteristics, including proliferation, migration and invasion.^29^ Another study reported that lncRNA DGCR promotes cancer cell stemness characteristics targeting the miR‐330‐5p/CD44 axis in NSCLC.^30^ In addition, lncGata6 maintains intestinal cell stemness and promotes tumour occurrence and progression in colorectal cancer.^31^ Silencing of LncRNA n339260 reduces the stemness of HCC cells.^32^ Zhang reported that lncRNA FEZF1‐AS1 regulates breast cancer stem cells by sponging miR‐30a, which targets Nanog.^33^

Several studies have indicated that lncRNA SPRY4‐IT1 is associated with the progress of breast cancer. LncRNA SPRY4‐IT1 is highly expressed in breast cancer cells, and N‐terminal polypeptide derived from viral macrophage inflammatory protein Ⅱ (NT21MP) inhibits the biological functions of breast cancer cells through lncRNA SPRY4‐IT1.^34^ Another study has reported that proliferation is significantly suppressed when SPRY4‐IT1 is knocked down in breast cancer cells by targeting ZNF703.^17^ However, it is not clear how SPRY4‐IT1 affects the stemness of breast cancer cells.

Transcription factor 7‐like 2 (TCF7L2), also called TCF4, affects tumour development because TCF7L2 plays a key role in the Wnt/β‐catenin signalling pathway.^35^ The Wnt/β‐catenin signalling pathway promotes cell proliferation and stem cell self‐renewal.^36^ Activation of the Wnt/β‐catenin signalling pathway maintains stemness of breast cancer cells.^37^ Thus, TCF7L2 mediates cell proliferation and stemness of breast cancer cells via the Wnt/β‐catenin signalling pathway.^23^

The proposed ceRNA (competing endogenous RNA) hypothesis suggests that crosstalk may exist among RNAs, thereby influencing biological processes independently of protein translation.^38^ Our study was based on the ceRNA hypothesis, in which SPRY4‐IT1 is directly bound to miR‐6882, thereby targeting TCF7L2. Therefore, we revealed that miR‐6882 is a new tumour suppressor‐miR in breast cancer that inhibits stemness characteristics through directly targeting TCF7L2 and further affecting the Wnt/β‐catenin signalling pathway.

**Figure 6:**
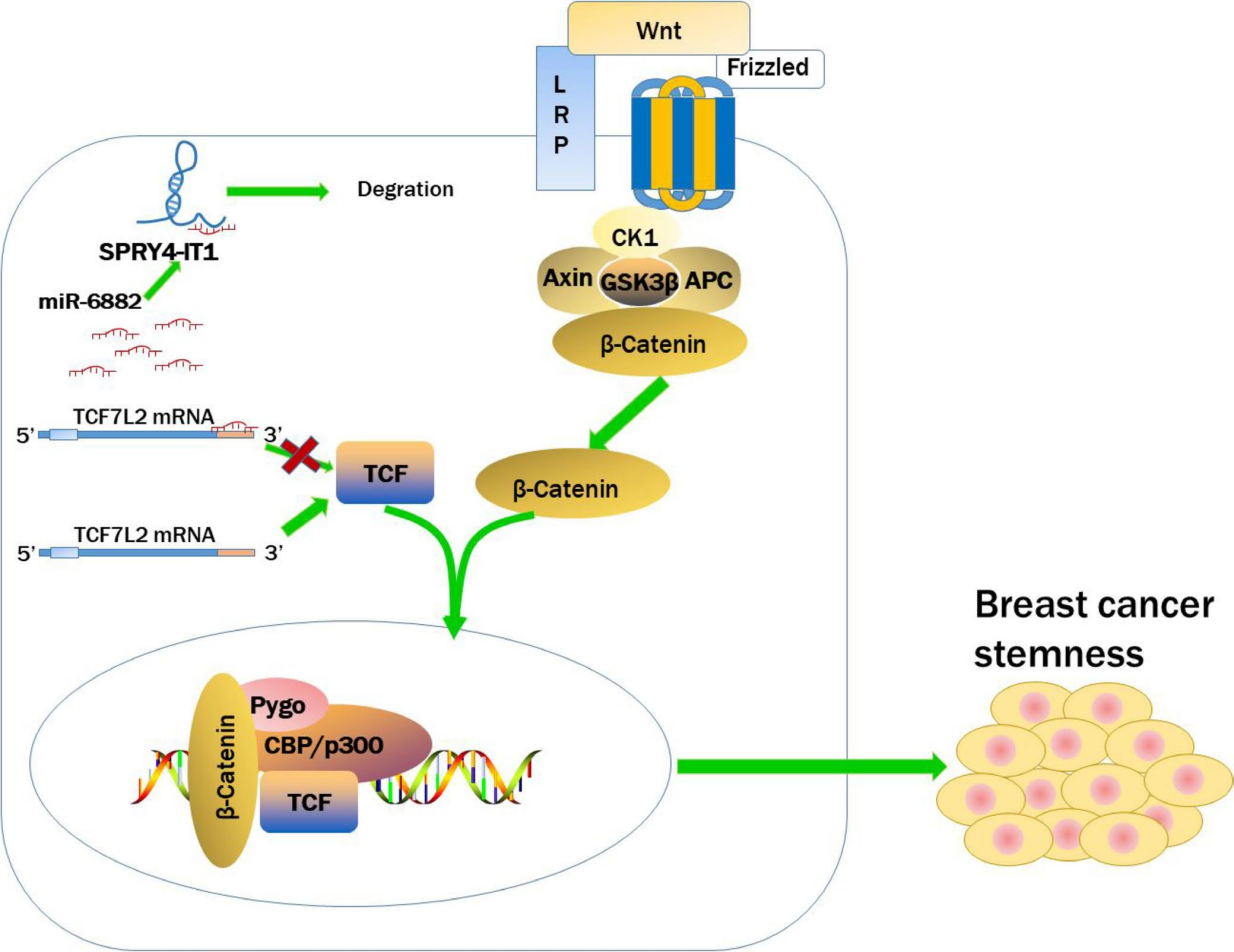
A schematic model indicating that SPRY4‐IT1 competitively binds to miR‐217 and TCF7L2, consequently regulating the Wnt/β‐catenin signalling pathway to promote stemness of breast cancer cells

Our study first indicated that SPRY4‐IT1 promoted breast cancer cell stemness. We found that SPRY4‐IT1 was highly expressed in MCF7 CSCs compared to MCF7 cells, which was further verified by data from TCGA. Next, we overexpressed SPRY4‐IT1 in MCF‐7 cells and knocked down SPRY4‐IT1 in MCF‐7 CSCs, which demonstrated that SPRY4‐IT1 promoted the stemness of breast cancer. We then verified that SPRY4‐IT1 is directly bound to miR‐6882 and that miR‐6882 was targeted to TCF7L2, which negatively regulated its expression. We also confirmed in vivo that SPRY4‐IT1 promoted the stemness and self‐renewal of BCSCs. These data suggested that SPRY4‐IT1 promotes stemness of breast cancer cells by targeting miR‐6882 through the Wnt/β‐catenin signalling pathway (see the proposed model in Figure 6). In our study, however, we only used MCF‐7 and T47D cell lines as experimental subjects. MCF‐7 and T47D are two types of human luminal A breast cancer cells.^39^ Our study only focused on luminal A breast cancer and did not include all types of breast cancer. MCF‐7, T47D, BT474, MDA‐MB‐436 and JIMT1 cells can be reproduced in long‐term mammosphere cultures, but MDA‐MB‐231 and 468 cells are unable to form long‐term mammosphere cultures.^40^ In the future, we will continue to investigate if SPRY4‐IT1 promotes stemness, cell proliferation and self‐renewal in other types of breast cancer cells.

Overall, the present study demonstrated that SPRY4‐IT1 is related to the stemness of breast cancer cells through miR‐6882 via the Wnt/β‐catenin signalling pathway.

## CONFLICTS OF INTEREST

The authors confirm that there are no conflicts of interest.

## AUTHOR CONTRIBUTIONS

Xinyue Song, Xiaoxue Zhang, Lin Zhao and Minjie Wei conceived and designed the project. Xinyue Song, Ming Zhang and Longyang Jiang designed and supervised experiments conducted in the laboratories. Xinyue Song, Xiaoxue Zhang, Xinnan Wang, Lianze Chen and Ang Zheng performed experiments and/or data analyses. Xiaoxue Zhang, Lin Zhao and Minjie Wei contributed reagents/analytic tools and/or grant support. Xinyue Song and Xiaoxue Zhang wrote the paper. All authors discussed the results and commented on the manuscript.

## Data Availability

The data that support the findings of this study are available from the corresponding author upon reasonable request.
