# Supplementary material for: LncRNA SPRY4‐IT1 regulates breast cancer cell stemness through competitively binding miR‐6882‐3p with TCF7L2
Source: J Cell Mol Med. 2019 Nov 17;24(1):772–84. doi: 10.1111/jcmm.14786 (PMC6933354; doi:10.1111/jcmm.14786)
Supplement: Supplementary file 1 [file JCMM-24-772-s001.docx]

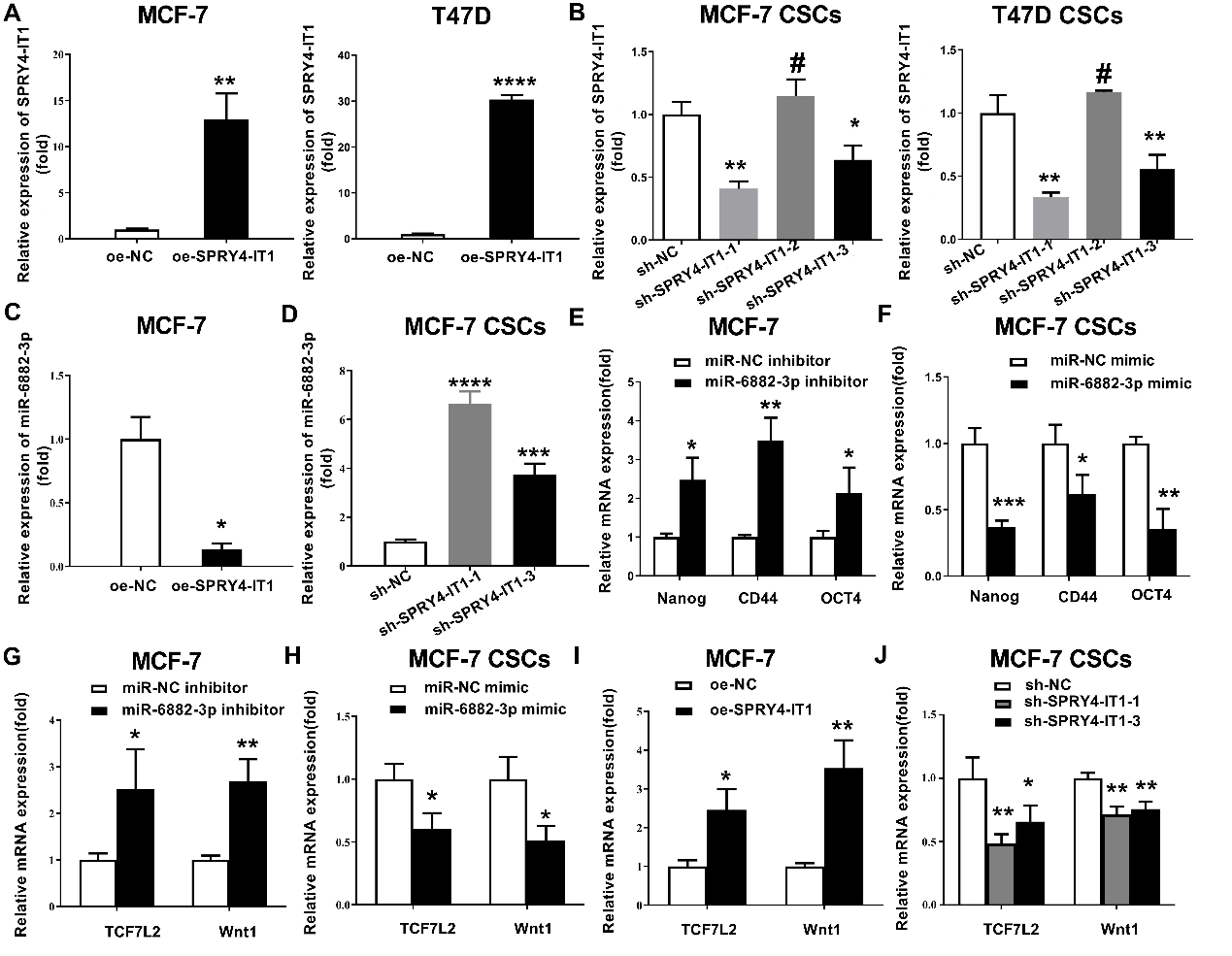


**Figure.S1** SPRY4-IT11 acted as a ceRNA for miR-6882-3p, related to Fig. 4. **A** SPRY4-IT1 expression was detected by qRT-PCR in SPRY4-IT1 overexpressing MCF-7 and T47D cells. **B** SPRY4-IT1 expression was detected by qRT-PCR in silencing SPRY4-IT1 of MCF-7 and T47D CSCs. **C** miR-6882-3p was detected by qRT-PCR in SPRY4-IT1 overexpressing MCF-7 and T47D cells. **D** miR-6882-3p was detected by qRT-PCR in silencing SPRY4-IT1 of MCF-7 and T47D CSCs. **E** Expression of stemness markers (Nanog, CD44 and OCT4) was detected by qRT-PCR in MCF-7 which inhibited miR-6882-3p. **F** Expression of stemness markers (Nanog, CD44 and OCT4) was detected by qRT-PCR in MCF-7 CSCs which overexpressed miR-6882-3p. **G** Wnt1/β-catenin signaling pathway-related mRNA expression (TCF7L2, Wnt1) was measured by qRT-PCR after inhibition of miR-6882-3p, or miR-NC in MCF-7 cells. **H** Wnt1/β-catenin signaling pathway-related mRNA expression (TCF7L2, Wnt1) was measured by qRT-PCR after overexpression of miR-6882-3p, or miR-NC in MCF-7 CSCs. **I** Wnt1/β-catenin signaling pathway-related mRNA expression (TCF7L2, Wnt1) was measured by qRT-PCR after overexpression of SPRY4-IT1 in MCF-7 cells. **J** Wnt1/β-catenin signaling pathway-related mRNA expression (TCF7L2, Wnt1) was measured by qRT-PCR after knockdown of SPRY4-IT1 in MCF-7 CSCs.


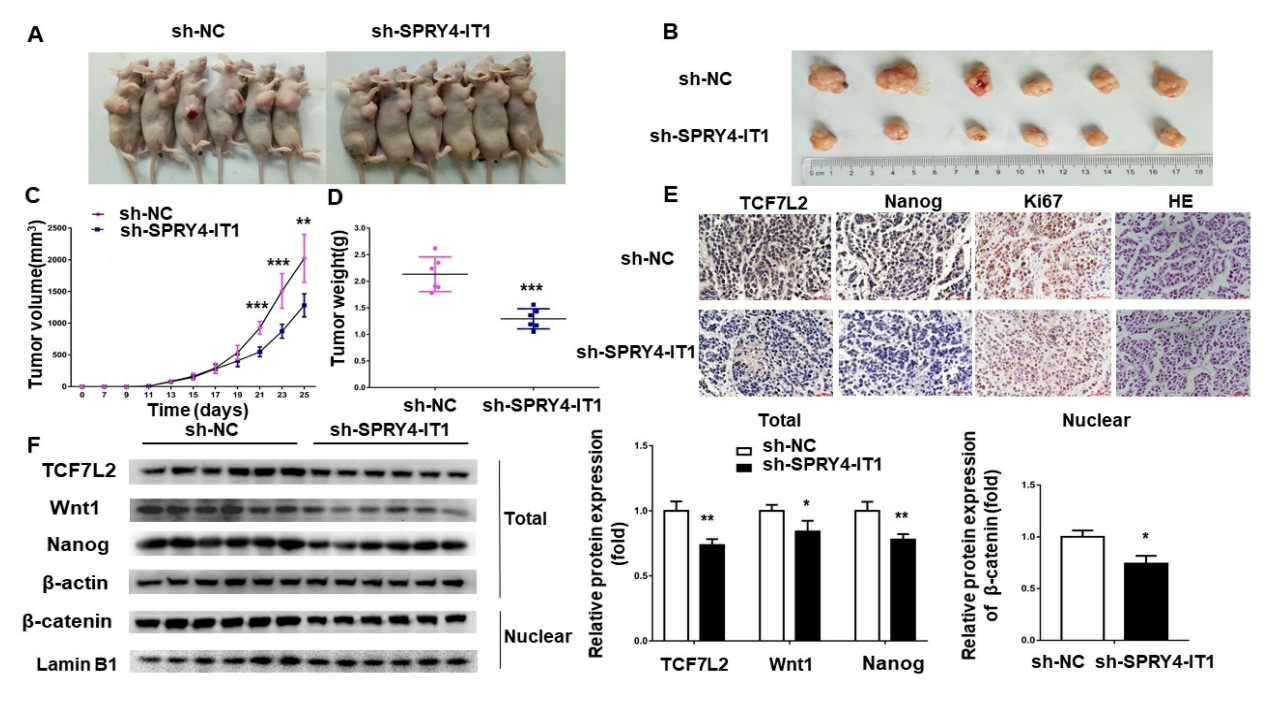


**Figure.S2** SPRY4-IT1 maintains the stemness of BCSCs in vivo. A Subcutaneous tumor was taken in SPRY4-IT1 knockdown (sh-SPRY4-IT1) group and negative control group. B Images of sh-SPRY4-IIT1 MCF7 tumor tissues. C Average tumor volumes were measured in xenograft mice every two days. D Images of average tumor weight at the end of indicated treatment. E Immunohistochemistry analysis of TCF7L2, Nanog and Ki-67 protein levels in tumor tissues formed from SPRY4-IT1 knockdown or control group. Original magnification, ×400. Scale bars, 50 μm. F Wnt1/β-catenin signaling pathway-related protein expression (TCF7L2, Wnt1, β-catenin (Nuclear)) and Nanog were measured by western blot in sh-NC and sh-SPRY4-IT1 groups. Data are presented as the mean ± SD of three independent experiments performed in triplicate. **P* < 0.05, ***P* < 0.01, ****P* < 0.001, *****P* < 0.0001.

**Supplementary Tables**

**Table S1.** The sequences for primers used in the study

| **Name** | **Sequences** |
| --- | --- |
| *Primers for qRT-PCR* | |
| U6-F  U6-R | 5’-TGGCACCCAGCACAATGAA-3  5’-CTAAGTCATAGTCCGCCTAGAAGCA-3’ |
| SPRY4-IT1-F | 5’-AGCCACATAAATTCAGCAGA-3’ |
| SPRY4-IT1-R | 5’- CGATGTAGTAGGATTCCTTTCA-3’ |
| OCT4-F  OCT4-R  C-MYC-F  C-MYC-R | 5′-CTTCGCAAGCCCTCATTTC-3′  5′-GAGAAGGCGAAATCCGAAG-3′  5′-GGCTCCTGGCAAAAGGTCA-3′  5′-CTGCGTAGTTGTGCTGATGT-3′ |
| Nanog-F | 5′-TTTGTGGGCCTGAAGAAAACT-3′ |
| Nanog-R | 5′-AGGGCTGTCCTGAATAAGCAG-3′ |
| SOX2-F | 5′-GCCGAGTGGAAACTTTTGTCG-3′ |
| SOX2-R | 5′-GGCAGCGTGTACTTATCCTTCT-3′ |
| CD44-F | 5′-AAGGTGGAGCAAACACAACC-3′ |
| CD44-R | 5′-TCGACTGTTGACTGCAATGC-3′ |
| TCF7L2-F | 5′-CTCCTCATCAATTGCACAGC-3′ |
| TCF7L2-R | 5′-GGAGCTGTGGGAATGTAACC-3′ |
| Wnt1-F | 5′-ACGTAGCCTCCTCCACGAACCTGC-3′ |
| Wnt1-R | 5′-CGCATCTCGGAGAATACGGTCG-3′ |
| β-actin -F | 5′-CTGGCCGGGACCTGACT-3′ |
| β-actin R | 5′-TCCTTAATGTCACGCACGATTT -3′ |
| *Sequence of SPRY4-IT- RNAi* | |
| SPRY4-IT1-RNAi-1 | AGCCACATAAATTCAGCAGAG |
| SPRY4-IT1-RNAi-2 | GGGGTTATAATAGGGAAGATA |
| SPRY4-IT1-RNAi-3 | GTGCTGAGGGGTTCTTAAATA |
| *Primers for SPRY4-IT1 ISH* | 5’- Dig- AATTTATGTGGCTGACAAAGGA -Dig-3’ |

Abbreviations: qRT-PCR, Quantitative real-time PCR; ISH, in situ hybridization; F, forward primer; R, reverse primer.

**Table S2.** Antibodies used for IHC and WB

| **Antibody** | **Company/Provider** | **Dilution ratio** |
| --- | --- | --- |
| anti-human TCF7L2 | Cell Signaling Technology | 1:1000 |
| anti-human β-catenin | Cell Signaling Technology | 1:1000 |
| anti-human Nanog | Cell Signaling Technology | 1:1000 |
| anti-human Wnt1 | ABclonal | 1:1000 |
| anti-human SOX2 | Cell Signaling Technology | 1:1000 |
| anti-human OCT-4 | Cell Signaling Technology | 1:1000 |
| anti-human C-MYC | Cell Signaling Technology | 1:1000 |
| anti-human β-actin | BOSTER | 1:1000 |
| goat anti-mouse IgG | EARTHOX Life Science | 1:10000 |
| goat anti-rabbit IgG | EARTHOX Life Science | 1:10000 |
| anti-human TCF7L2(IHC) | Cell Signaling Technology | 1:200 |
| anti-human Nanog(IHC) | Cell Signaling Technology | 1:200 |
| anti-human Ki-67(IHC) | abcam | 1:200 |
